# Fertility and Viability of Hybrid Offspring Imply the Absence of Major Postzygotic Isolation Between Two *Reticulitermes* Termite Species

**DOI:** 10.3390/insects17030350

**Published:** 2026-03-23

**Authors:** Jia Wu, Yonghui Wang, Bei Du, Xiaolan Wen

**Affiliations:** 1 College of Pharmacy, Shaanxi Institute of International Trade and Commerce, Xianyang 712046, China; yonghuiwang2025@163.com (Y.W.); 18082565686@163.com (B.D.); 2 Applied Research Center for Life Science, Xi’an International University, Xi’an 710077, China; 3 College of Medicine, Shaanxi Institute of International Trade and Commerce, Xianyang 712046, China; 4 School of Preclinical Medicine, Guangxi Medical University, Nanning 530021, China

**Keywords:** hybridization, reproductive isolation, termites, genetic dilution

## Abstract

(1) Reproductive isolation serves as a critical mechanism that prevents interspecific hybridization among closely related species, thereby preserving species integrity. In termites, hybridization between certain closely related species can overcome prezygotic isolation and produce offspring. However, whether these hybrids can overcome postzygotic barriers remains substantially underexplored. (2) This investigation conducted a comparative analysis of reproductive output (egg production and offspring count), physiological traits (body weight), functional characteristics (locomotor capacity), and hybrid fertility between the hybrid colony that was established by *Reticulitermes flaviceps* and *R. chinensis* and the conspecific pairing colonies of *R. flaviceps* and *R. chinensis*, respectively. (3) The results showed that hybrid colonies laid significantly more eggs and produced significantly more larvae than conspecific colonies. The hybrid offspring showed no decline in weight and locomotor capacity. Furthermore, the hybrid offspring maintained balanced sex ratios with unimpaired caste differentiation and fertility in both sexes. (4) These results demonstrate that the prezygotic reproductive isolation mechanism is incomplete between two closely related termite species, and there is a risk of forming hybrid populations. This work not only provides a theoretical basis for monitoring the risk of hybrid populations in termite management but also offers new insights into the evolution of reproductive isolation and speciation in social insects.

## Simple Summary

Reproductive isolation is essential for maintaining species integrity by limiting interspecific hybridization. In termites, some closely related species can bypass prezygotic isolation and produce hybrids, yet postzygotic barriers remain poorly understood. This study compared reproductive output, body weight, locomotor ability, and hybrid fertility between the hybrid colonies of *Reticulitermes flaviceps* and *R. chinensis* and their conspecific colonies. The hybrid colonies displayed higher egg and larval production, with no reduction in body weight or locomotor capacity. The hybrid offspring showed normal sex ratio, caste differentiation, and fertility. These findings indicate an incomplete prezygotic reproductive isolation between the two termites and a potential hybrid population formation. This work supports termite risk management and enriches the understanding of reproductive isolation and speciation in social insects.

## 1. Introduction

Interspecific hybridization constitutes a principal mechanism of gene flow between species and acts as a key evolutionary driver, which accelerates the speciation processes [1,2]. Previous studies have long held the view that interspecific hybridization was considered a rare occurrence in nature. Even if some cases of interspecific hybridizations have been reported, they were mostly concentrated in plants [3,4,5]. However, with advances in molecular biology and bioinformatics, as well as the increasing sophistication of research methodologies, recent studies have revealed that interspecific hybridization is ubiquitous across diverse animal lineages. Specifically, such hybridization has been documented in insects [6,7], amphibians [8,9], reptiles [10,11], fish [12,13], birds [14,15], and mammals [16]. These cases collectively challenge the traditional perception that interspecific hybridization is rare or plant-specific. While these studies predominantly emphasize interspecific gene exchange, a comparatively small amount of attention has been paid to the impact of reproductive barriers on the hybridization between species.

Reproductive isolation strictly restricts interspecific hybridization through multiple mechanisms: prezygotic isolation [17] and postzygotic isolation by hybrid inviability [18,19], hybrid sterility [20], and hybrid breakdown [21]. However, empirical observations have revealed that the ecological niches of two species—particularly in some closely related taxa—often lack clear boundaries [22]. Notably, their niches often exhibit partial overlap, such as a partial overlap in breeding seasons, similar breeding behaviors, and coexistence in the same geographical region, which provides favorable conditions for individuals of different species to encounter one another and reproduce, forming a hybrid zone [22,23]. Furthermore, within such hybrid zones, strong interspecific interactions enable some closely related species to overcome prezygotic isolation, thereby further facilitating interspecific gene exchange [24,25].

In closely related species, although they undergo independent selection and their mutation and heredity are adapted to their ecological niches [26], genetic recombination and genetic structure remodeling remain incomplete due to the relatively short divergence time. When such closely related species are sympatric or re-encountered due to reasons like range expansion and migration, hybridization may occur [23,27]. For instance, the extensive hybridization between *Coptotermes formosanus* (Formosan subterranean termite) and *C. gestroi* (Asian subterranean termite) has been reported in southern Florida’s invasive populations [23,28,29]; the sympatric populations of termite species *Neotermes corniger* (Florida dampwood termite) and *N. ephratae* (Southeast Asian dampwood termite) successfully produce hybrid offspring despite a phylogenetic divergence [30]. Our previous research also found that the closely related agricultural pests, *Reticulitermes flaviceps* Oshima and *R. chinensis* Snyder, share overlapping habitats, synchronized dispersal seasons, similar sex pheromones, and comparable reproductive behaviors [22,31]. The interspecific hybrids between *R. chinensis* and *R. flaviceps* can produce viable offspring [31]. However, it remains unclear whether F1 hybrids that are derived from these closely related termite species attain reproductive maturity, i.e., whether they can achieve complete developmental viability and overcome post-mating reproductive isolation and produce viable offspring, thereby establishing persistent hybrid lineages.

To explore this question, we compared the number and sex ratio of the F1 offspring, as well as their body weight and locomotor capacity, between the hybrid pairing colonies (including *R. chinensis* ♂-*R. flaviceps* ♀ and *R. chinensis* ♀-*R. flaviceps* ♂) and the conspecific pairing colonies (*R. flaviceps* ♂-*R. flaviceps* ♀ and *R. chinensis* ♀-*R. chinensis* ♂) of *R. chinensis* and *R. flaviceps*. Additionally, this study examined whether the F1 workers from hybrid pairing colonies can develop into supplementary reproductives and whether these F1 reproductives can backcross with the original reproductives to produce F2 individuals. This will provide insight into the evolution of reproductive isolation and speciation in termites.

## 2. Materials and Methods

### 2.1. Termites

The closely related species *R. flaviceps* and *R. chinensis* are consistently different in terms of morphology, biology, and molecular genetics [22,32]. In this study, both species were collected in March 2021 from Nanjing (Jiangsu Province) and Xi’an (Shaanxi Province), just prior to their swarming season. Sections of termite-infested logs containing groups of termites (including last-instar nymphs and/or alates) were carefully transferred to the laboratory and placed individually in plastic boxes (70 cm × 60 cm × 50 cm), which were covered with fine nylon mesh. The boxes were maintained at 16 °C until the experiments commenced. Prior to each experiment, the plastic boxes were moved to a room set at 30 °C to simulate the conditions of their initial dispersal flight and promote swarming. After swarming, the sequential reproductive behaviors of termites included wing shedding, tandem behavior, pair formation, nest construction, and finally mating [33,34]. To prevent pre-experimental mating, alates from each colony were separated by sex and kept with nestmates in Petri dishes containing a piece of moist filter paper and sawdust, which served as water and food sources, respectively. Within 24 h, approximately half of the individuals shed their wings spontaneously to become dealates. We then removed the remaining alates and collected the dealates for subsequent experiments.

### 2.2. Pairing Experimental Treatments

Six colonies of *R. flaviceps* were labeled A1–A6, and six colonies of *R. chinensis* were labeled B1–B6. Alates from the *R. flaviceps* colonies were paired with alates from the *R. chinensis* colonies to form six cross-colony combinations: A1B1, A2B2, A3B3, A4B4, A5B5, and A6B6. Two types of hybrid pairing were established: one consisting of a male *R. chinensis* paired with a female *R. flaviceps* (*Rc* ♂-*Rf* ♀) and the other consisting of a female *R. chinensis* paired with a male *R. flaviceps* (*Rc* ♀-*Rf* ♂). Meanwhile, the conspecific pairing colonies, which comprised a couple of *R. flaviceps* (*Rf* ♂-*Rf* ♀) and a couple of *R. chinensis* (*Rc* ♂-*Rc* ♀), were established as the control. For each pairing type, 24 replicates were established, with each pairing maintained in a Petri dish (ø = 6 cm) lined with moistened filter paper, in constant darkness, and in a temperature range of 20–26 °C. Water was provided as necessary. Observations were conducted every two days, during which the number of eggs in the first batch and the larvae that were produced were carefully recorded. After the first batch of eggs had hatched completely, half of the 24 colonies (12 colonies) from each pairing type were randomly selected to measure the fresh weight, locomotor behavior, and sex ratio of the offspring. The remaining 12 colonies were continuously maintained for subsequent examination of the fertility of the hybrid offspring.

### 2.3. Physiological and Behavioral Characteristics

Considering the potential for hybrid breakdown in interspecific hybridization, we measured the physiological and behavioral characteristics of workers that were derived from different pairing types. Following the caste differentiation in the colony, the fresh weight of the third instar and older workers was measured using an analytical balance (Sartorius Quintix Pro, Göttingen, Germany). Each treatment consisted of eight initial laboratory-reared pairing colonies. For each colony, no fewer than six workers were weighed individually, and their weights were recorded separately (detailed sample sizes are provided in Table 1).

To compare the locomotor behavior of workers from conspecific and hybrid pairings, individual workers were placed separately into an observation arena consisting of a Petri dish filled with moistened paper. A high-definition (HD) camera (Nikon D7000 with 60 mm lens, Tokyo, Japan) was positioned above the arena for behavioral recording. The video was captured at a rate of 25 frames per second (fps) for each individual, starting 5 min after the worker was placed. A total of 30 replicates were performed for each worker type. The locomotor activity was recorded over a 5 min period, and the movement trajectory, distance and velocity during walking were quantified using the EthoVision video-tracking system (EthoVision XT 15, Noldus, Wageningen, The Netherlands).

### 2.4. The Gender Ratio in Hybrid Offspring

To determine whether the sex ratio of hybrid F1 individuals is normal, we employed male-specific primers to analyze the sex ratio of F1 individuals from conspecific and hybrid pairings. For the sex ratio analysis, 4 colonies of each type were used, and 6 individuals were randomly selected from each colony. DNA was extracted from each individual using a TIANamp Genomic DNA Kit (Tian Gen Biotech Co., Ltd., Beijing, China) according to the manufacturer’s instructions. A PCR amplification was performed using a male-specific sequence (Forward 5′-ACGGCCACTCTTCATTCTCT-3′ and Reverse 5′-ATGTGTTGAAATGGGGCCAC-3′) and gene COII. The mitochondrial gene COII (Forward 5′-CAGATAATGGCATTGGTTT-3′ and Reverse 5′-GTTTAAGAGCACATTACTA-3′) was used as a positive control for DNA quality, and all primers were synthesized by Invitrogen Trading Co., Ltd. (Shanghai, China). The sex of the F1 individuals was estimated using the following standards: (1) the individuals that can simultaneously amplify the target fragment with male-specific sequences and the COII gene are male individuals, and (2) the individuals that can amplify electrophoresis bands with the COII gene but not with male-specific sequences are female individuals.

### 2.5. Caste Differentiation and Reproductive Capacity of Hybrid Offspring

The hybrid offspring sterility and hybrid breakdown are primary mechanisms that establish effective reproductive isolation between species. To assess the fertility of the F1 individuals in hybrid colonies, the following treatments were conducted on the 2-year-old laboratory-reared hybrid colonies of *R. flavicepes* and *R. chinensis*: (a) the removal of the primary queen; (b) the removal of the primary king; and (c) the simultaneous removal of both the primary queen and king. The sample sizes per treatment are provided in Table 2. After the removal of the primary reproductives, a successful differentiation of workers into secondary reproductives in hybrid colonies indicates that the F1 hybrids possess the capacity for caste differentiation. If these secondary reproductives can lay eggs and produce viable larvae, it confirms that the F1 hybrids are functionally fertile. We conducted monthly observations, and the number of colonies in which workers developed into secondary reproductives and the number of workers that produced F2 individuals were recorded.

### 2.6. Statistical Analysis

Statistical analyses of the data were performed using SPSS V21 (IBM Corp., Armonk, NY, USA). All the results are presented as mean values ± the standard error of the mean (means ± SEM). One-way analysis of variance (ANOVA) was used to compare the data on the number of eggs laid, number of larvae, body weight of the third instar and older workers, and locomotor traits (locomotor speed and distance) across different colony pairing types in both termite species. The differences in mean values between treatments were analyzed using Tukey’s honestly significant difference (HSD) test, with significance defined at *p* < 0.05. The sex ratio differences between conspecific and hybrid pairings were evaluated using chi-square (χ^2^) tests, with *p* < 0.05 indicating significance.

## 3. Results

**Figure 1:**
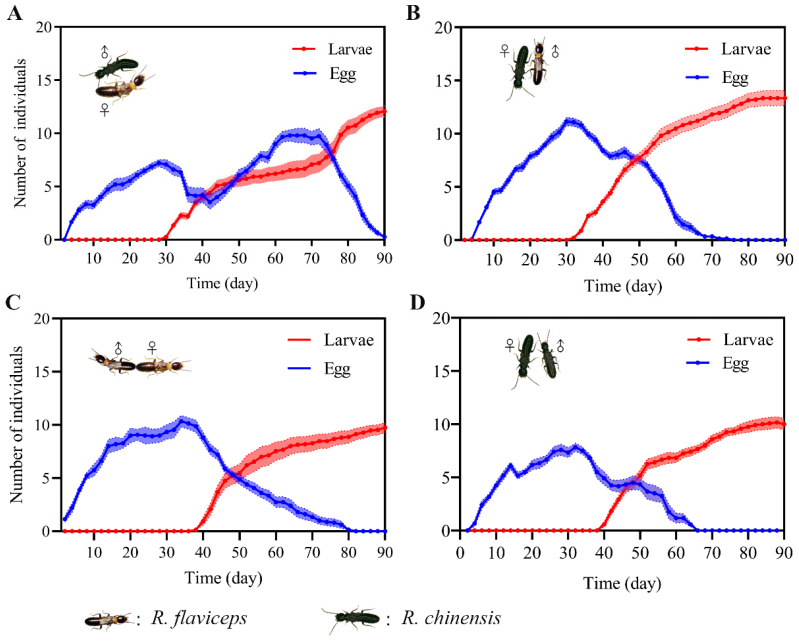
The changes in egg production and larvae in different pairing-type colonies. (**A**) A colony established through hybridization between a male *R. chinensis* and a female *R. flaviceps*; (**B**) a colony established through hybridization between a female *R. chinensis* and a male *R. flaviceps*; (**C**) a colony established by a pair of *R. flaviceps*; and (**D**) a colony established by a pair of *R. chinensis*. The shaded areas represent the mean individuals ± the standard error.

Our results showed that hybrid pairs began laying eggs immediately following pair formation, exhibiting temporal patterns that are analogous to those of conspecific pairings (Figure 1). A significant variation in the number of eggs laid was observed between colony compositions under laboratory conditions (One-way ANOVA: *F* _(3,119)_ = 8.98, *p* < 0.001). Specifically, the number of eggs laid in conspecific pairing colonies including both *R. chinensis* (*Rc* ♂-*Rc* ♀) and *R. flaviceps* (*Rf* ♂-*Rf* ♀) were comparable to that observed in the egg production of the hybrid colony that was established by an *R. chinensis* male and an *R. flaviceps* female (*Rc* ♂-*Rf* ♀) (Tukey’s HSD, *Rc* ♂-*Rc* ♀ vs. *Rc* ♂-*Rf* ♀: *p* = 0.168 and *Rf* ♂-*Rf* ♀ vs. *Rc* ♂-*Rf* ♀: *p* = 0.677). However, the number of eggs laid in colonies where *R. flaviceps* males were paired with *R. chinensis* females (*Rf* ♂-*Rc* ♀) was significantly higher than the number of eggs laid in the conspecific colonies, including both *R. chinensis* (*p* = 0.001) and *R. flaviceps* (*p* < 0.001).

Regarding the hatching period of eggs, no statistically significant differences were observed between the conspecific pairings of *R. flaviceps* and *R. chinensis* (*p* = 0.87). Similarly, the reciprocal hybrid pairings (*Rf* ♂-*Rc* ♀ vs. *Rc* ♂-*Rf* ♀) showed comparable hatching periods (*p* = 0.95). However, the hybrid pairing colonies collectively exhibited substantially shorter hatching periods compared to the conspecific pairing colonies (Figure 1, One-way ANOVA: *F* _(3,119)_ = 7.52, *p* < 0.0001).

There were more larvae produced in hybrid *Rc* ♂-*Rf* ♀ and *Rf* ♂-*Rc* ♀ colonies compared with the conspecific pairing colonies of *R. chinensis* (*Rc* ♂-*Rc* ♀) and *R. flaviceps* (*Rf* ♂-*Rf* ♀) (Figure 1, One-way ANOVA: *F* _(3,119)_ =14.32, *p* < 0.0001; Tukey’s HSD, *Rc* ♂-*Rc* ♀ vs. *Rc* ♂-*Rf* ♀: *p* = 0.0002; *Rf* ♂-*Rf* ♀ vs. *Rc* ♂-*Rf* ♀: *p* = 0.0047; *Rc* ♂-*Rc* ♀ vs. *Rf* ♂-*Rc* ♀: *p* < 0.0001; and *Rf* ♂-*Rf* ♀ vs. *Rf* ♂-*Rc* ♀: *p* < 0.0001), with no significant difference observed between the two conspecific pairing colonies (*p* = 0.68) and no significant difference observed between the two hybrid pairing colonies (*p* = 0.59). These findings suggest that *R. flaviceps* and *R. chinensis* heterospecific pairing colonies possess a greater reproductive capacity compared to conspecific pairing colonies.

**Figure 2:**
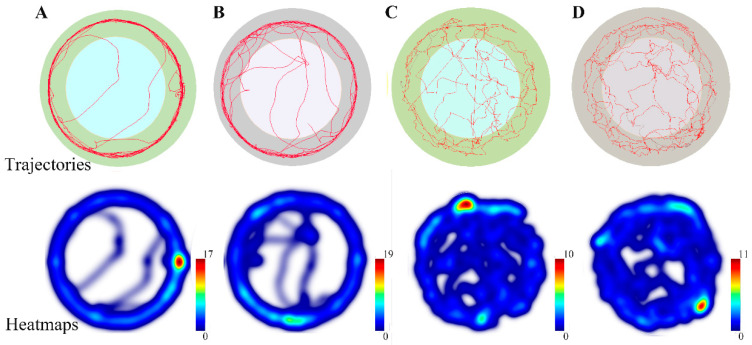
The motion trajectories and heatmaps of the offspring produced in different pairing-type colonies. (**A**) The hybrid individuals of male *R. chinensis* and female *R. flaviceps*; (**B**) the hybrid individuals of female *R. chinensis* and male *R. flaviceps*; (**C**) the individuals produced by *R. flaviceps* pairs; (**D**) the individuals produced by *R. chinensis* pairs. The movement trajectories directly reflect an individual’s activity level, movement range, and velocity; longer and more continuous trajectories represent a stronger locomotor ability and a higher activity level. The trajectory heatmaps visualize the spatial density and dwell on the time of trajectories, thus intuitively revealing the individuals’ activity patterns, range utilization, spatial preferences, and path choices.

**Figure 3:**
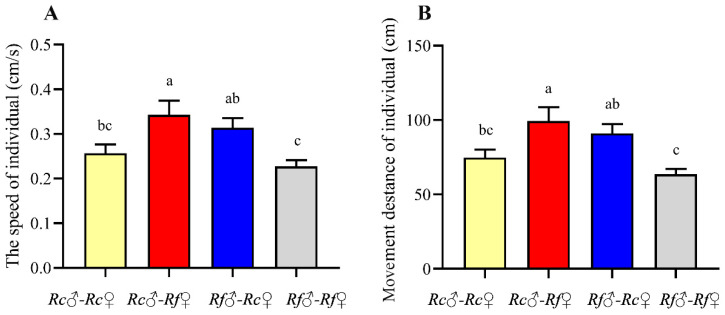
The motion speed and distance of the offspring produced in different pairing-type colonies. (**A**) The speed of the individual and (**B**) the movement distance of the individual. Different letters show significant differences among treatments (*p* < 0.05).

We found that in two conspecific colonies, the workers of *R. flaviceps* were heavier. The workers of the hybrid colonies between *R. flaviceps* and *R. chinensis* showed comparable weight. However, interestingly, the weight of the workers produced by the hybridization between *R. flaviceps* and *R. chinensis* was just between the workers of *R. flaviceps* and *R. chinensis*, and there was no significant difference between the workers of *R. flaviceps* and *R. chinensis* (Table 1, One-way ANOVA: *F* _(3,31)_ = 3.24, *p* = 0.037). The movement trajectories of the workers produced via a hybrid between *R. flaviceps* and *R. chinensis* (*Rf*♂-*Rc* ♀ and *Rf* ♀-*Rc* ♂) were mainly distributed in the area covered by the wall of the Petri dish, with minimal exposure in the open area (Figure 2A,B). In contrast, the movement trajectories of workers in *R. flaviceps* and *R. chinensis* were relatively evenly distributed (Figure 2C,D). In addition, significant differences were also observed in the movement speed (Figure 3A, *F* _(3,68)_ = 5.31, *p* = 0.0024) and distance (Figure 3B, *F*_(3,68)_ = 6.12, *p* = 0.001) of workers among the different pairing types of colonies. These results showed that the workers produced by the hybridization of *R. flaviceps* and *R. chinensis* had stronger movement ability.

The data are shown as the means ± SEM, and the different letters indicate significant differences (Tukey’s HSD test, *p* < 0.05). *Rc* and *Rf* indicate *R. chinensis* and *R. flaviceps*, respectively.

**Figure 4:**
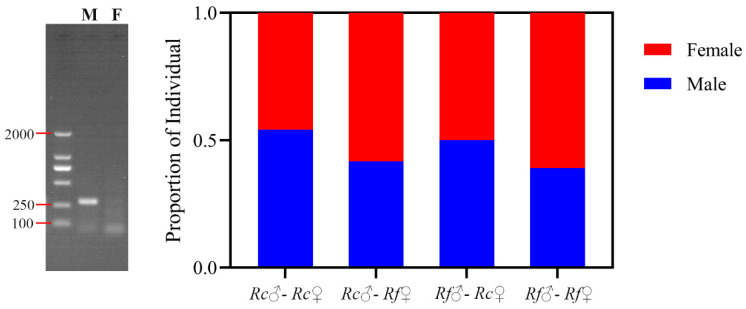
The sex ratio of F1 individuals in different pairing-type colonies. Through amplification with male-specific primers, PCR products from male individuals exhibit distinct amplification bands (290 bp), while female individuals show no bands. Similar to conspecific pairings, the hybrid pairings of *R. flaviceps* and *R. chinensis* produced offspring of both sexes, and no significant difference occurred in the sex ratios between the hybrid and conspecific offspring.

The sex-specific analysis revealed that the sex ratio of the offspring produced by *R. flaviceps* and *R. chinensis* colonies did not exhibit any bias towards either sex (Figure 4; *x*^2^ = 0.75, *df* = 1, *p* = 0.38). Similar to their conspecific colonies, hybrid colonies of *R. flaviceps* and *R. chinensis* (*Rf* ♂-*Rc* ♀ and *Rf* ♀-*Rc* ♂) produced offspring containing individuals of both sexes. Furthermore, there was no significant difference in the sex ratio between hybrid and conspecific offspring (Figure 4; *x*^2^ = 1.18, *df* = 3, *p* = 0.75). Therefore, it can be concluded that there is no sex bias or deviation from the expected sex ratio in the offspring of the hybrid colonies of *R. flaviceps* and *R. chinensis*.

**Figure 5:**
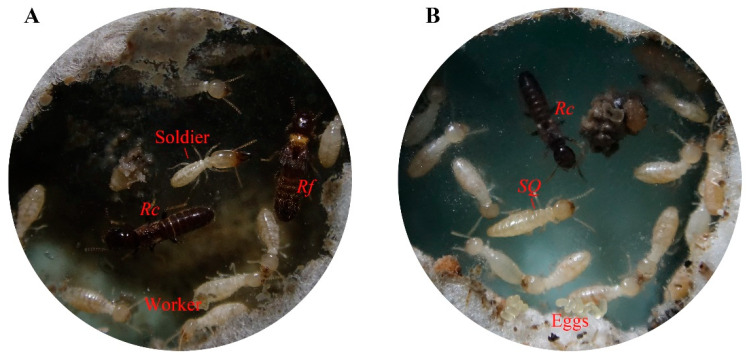
The hybrid colonies established by *R. flaviceps* and *R. chinensis*. (**A**) The soldiers in the hybrid colony and (**B**) the secondary queen in the hybrid colony. The workers in the hybrid pairing colonies of *R. flaviceps* and *R. chinensis* exhibited bipotential caste differentiation, developing into both soldier and secondary reproductive. *Rc* and *Rf* indicate *R. chinensis* and *R. flaviceps*, respectively; SQ, secondary queen.

Our results showed that workers in the hybrid pairing colonies of *R. flaviceps* and *R. chinensis* exhibited bipotential caste differentiation, developing into both soldiers (Figure 5A) and secondary reproductives (Figure 5B). Upon removal of the primary queen from the hybrid colonies, female hybrid workers underwent molting to become functional secondary queens. Analogously, the removal of the primary king in hybrid colonies triggered male workers to differentiate into secondary kings. Crucially, the simultaneous removal of both the primary queen and king resulted in caste-specific differentiation: the female and male workers developed into secondary queens and kings, respectively. These observations indicate preserved caste differentiation mechanisms in the hybrid colonies between *R. flaviceps* and *R. chinensis*. Notably, we conducted further breeding observations on the hybrid colonies, and the results showed that 75% of queen-removed colonies, 60% of king-removed colonies, and 65% of queen-and-king-removed colonies produced viable eggs and larvae (Table 2). These results demonstrate that secondary queens and kings derived from the hybrid workers maintained fertility, confirming that the offspring produced by the hybridization between *R. flaviceps* and *R. chinensis* are fertile.

## 4. Discussion

The ability of interspecific hybridization to produce viable offspring, the fertility of hybrid offspring, and the occurrence of hybrid breakdown are important criteria for evaluating postzygotic isolation between species [1,35]. In natural systems, prezygotic isolation typically prevents the formation of hybrid zygotes; even when limited hybridization occurs [17], developmental anomalies frequently manifest during early embryogenesis [18,19,25]. The hybrid individuals that survive often fail to attain sexual maturity [36]. Our study results confirmed that hybridization between *R. flaviceps* and *R. chinensis* produces viable offspring, which confirms the prior findings [22,31]. Compared with the conspecific pairings of *R. flaviceps* or *R. chinensis*, the interspecies hybrid colonies exhibit advantages in both quantity and quality, characterized by enhanced oviposition rates and elevated offspring production (Figure 1). These outcomes align with the observations in the experimental hybrid colonies between the Formosan subterranean termite *C. formosanus* and the Asian subterranean termite *C. gestroi* in southern Florida [23,28]. This elevated reproductive output provides evidence for quantitative heterosis (i.e., hybrid vigor) in termite hybrids.

Considering the growth and development of the hybrid offspring, their adaptability to the environment, and the potential for stable vertical transmission of hybrid genomes to subsequent generations, we quantified the body weight, locomotor ability, and sex ratio of the hybrid offspring. The results showed that hybrid offspring exhibited normal growth and differentiation without sexual dimorphism biases. This contrasts sharply with the documented asymmetries in other hybridization systems [6,25,30,37]. The hybridization of *R. flaviceps* and *R. chinensis* demonstrates symmetrical reciprocal compatibility, while *Nasutitermes corniger* and *N. ephratae* crosses are characterized by reciprocal directionality, which determines developmental competence [30]. The hybridization of *R. flaviceps* and *R. chinensis* also lacks the sex-specific developmental failures that are observed in the hybridization between *Formica polyctena* and *F. aquilonia* [6]. Notably, the hybrid offspring of *R. flaviceps* and *R. chinensis* exhibit enhanced locomotor speeds and a greater inclination to choose paths with available shelter. Measures such as increased locomotor speed, seeking appropriate shelters, and minimizing exposure in open areas have been demonstrated as effective strategies for evading predators in various organisms, including rodents, birds, and insects [38,39]. The heightened speed and reduced exposure of the hybrid offspring from *R. flaviceps* and *R. chinensis* indicate that they possess superior anti-predator strategies, which facilitate their adaptability to the environment within complex habitats. The hybrid offspring exhibit heterosis (such as strong locomotor ability and high stress resistance), which may lead to ecological invasiveness and practical pest control risks. This also reminds us that we need to monitor the distribution and spread of hybrid populations in termite control.

An important characteristic of the reproductive system in *Reticulitermes* colonies is that when primary kings/queens die or experience reproductive senescence, secondary reproductives replace them to assume reproductive functions, ensuring colony growth and persistence [33,34,40,41]. This characteristic extends to the hybrid colonies of *R. flaviceps* and *R. chinensis*. We found that artificial removal of parental reproductives induced the F1 hybrid workers (derived from *R. flaviceps*–*R. chinensis* crosses) to differentiate into secondary reproductives. Furthermore, these secondary reproductives demonstrated reproductive competence through two pathways: successful backcrossing with the primary reproductives of *R. flaviceps* or the *R. chinensis* that yielded viable offspring, and the mating between female and male secondary reproductives (F1 ♀ × F1 ♂) that produced F2 progeny. This confirms that the hybrid offspring between *R. flaviceps* and *R. chinensis* are fertile. Hybrid sterility typically arises from hybridization-induced chromosomal rearrangements that disrupt meiotic segregation [3,24]. However, as congeneric species with minimal phylogenetic divergence, *R. flaviceps* and *R. chinensis* undergo homoploid hybridization without ploidy changes [42]. This interspecific hybridization facilitates a sustained genetic introgression and the transfer of adaptive alleles between the two species lineages.

Divergence time may be one of the key factors influencing the postzygotic isolation between species [27,43]. The postzygotic isolation is often incomplete in recently diverged species, as they remain in the early stages of differentiation [27]. Although these species undergo independent selection, mutation, and genetic transmission that are adapted to their ecological niches, genomic recombination and gene structure remodeling remain incomplete due to their relatively short divergence times [16,26,27]. When such closely related species are sympatric, hybridization may occur, creating opportunities for interspecific gene flow. For instance, following the introduction of apples to North America, the apple maggot fly (*Rhagoletis pomonella*) underwent a host shift: some populations transitioned from their original host to the newly introduced apple, forming distinct host races. Owing to their relatively recent divergence, these populations can still interbreed and reproduce freely upon contact [26]. In contrast, for species with longer divergence times, even in the absence of premating isolation, hybrid offspring may suffer from hybrid breakdown or infertility, thereby preventing gene flow between species [44,45,46].

The subterranean termites *R. flaviceps* and *R. chinensis*, which diverged approximately 1.9–2.7 million years ago [47,48], exhibit a relatively long divergence time. Despite the observed heterotic advantages in the hybrid colonies that were established by *R. flaviceps* and *R. chinensis* under controlled conditions, and the confirmed fertility of F1 neotenic reproductives, definitive conclusions regarding the absence of postzygotic reproductive isolation remain premature. The key limitations necessitate caution. (1) While the F1 hybrid workers differentiate into neotenic reproductives, alate production is critical for natural dispersal and gene flow, and it remains unverified. (2) Reproductive isolation is a multifactorial process, where singular advantages (e.g., enhanced fecundity, locomotor capacity, predator avoidance) may not comprehensively offset cumulative postzygotic barriers. (3) The reproductive traits of replacement and backcrossing extend to the hybrid colonies, enabling a sustained gene flow between parental lineages. Continuous backcrossing may induce genetic dilution, potentially eliminating genetic components of one ancestor and causing hybrid populations to revert entirely to the gene pool of another parent [22]. Consequently, while hybridization facilitates interspecific genetic exchange, the concomitant processes of replacement and backcrossing counteract differentiation. This process effectively suppresses hybrid speciation through genetic homogenization, ultimately reinforcing postzygotic reproductive isolation. This represents a plausible framework that was derived from our findings and informed by the reproductive biology of *Reticulitermes* taxa. However, this proposition requires rigorous empirical validation through further investigation.

## Figures and Tables

**Table 1:** The weight of workers in different pairing-type colonies (unit: 10^−3^ g).

Pairing Type of Colonies	Number of Colonies	Number of Individuals	Weight
*Rc* ♀-*Rc* ♂	8	59	0.807 ± 0.029 ^b^
*Rf* ♀-*Rf* ♂	8	56	0.992 ± 0.035 ^a^
*Rc* ♀-*Rf* ♂	8	61	0.927 ± 0.049 ^ab^
*Rf* ♀-*Rc* ♂	8	58	0.917 ± 0.051 ^ab^

*Rc* and *Rf* indicate *R. chinensis* and *R. flaviceps*, respectively. Different letters show significant differences among treatments (*p* < 0.05).

**Table 2:** The proportion of hybrid colonies with neotenics and F2 individuals after removing the primary reproductive *.

Types of Hybrid Colony	Treating Types for the Primary Reproductives	The Proportion of Colonies with Neotenics	The Proportion of Colonies with F2 Individuals
*Rc* ♀ *× Rf* ♂	Removed king (n = 10)	100%	80%
	Removed queen (n = 10)	100%	70%
	Removed king and queen (n = 10)	90%	60%
*Rf* ♀ *× Rc* ♂	Removed king (n = 12)	100%	75%
	Removed queen (n = 12)	100%	75%
	Removed king and queen (n = 11)	91%	63.6%

* n: the number of samples. *Rc* and *Rf* indicate *R. chinensis* and *R. flaviceps*, respectively.

## Data Availability

The original contributions presented in this study are included in the article. Further inquiries can be directed to the corresponding authors.
